# MRI-derived radiomics model for predicting intratumoral tertiary lymphoid structures in soft tissue sarcoma

**DOI:** 10.1186/s13244-025-02086-3

**Published:** 2025-09-23

**Authors:** Tongyu Wang, Liping Wang, Hao Zhong, Yuanyuan Zong, Feng Hou, Hexiang Wang, Ruizhi Zhou, Song Gao, Xianglong Shi, Jiangfei Yang, Dapeng Hao

**Affiliations:** 1 Department of Radiology, The Affiliated Hospital of Qingdao University, Qingdao, 266003 Shandong China; 2 Department of Radiology, Yantai Yuhuangding Hospital, Affiliated Hospital of Qingdao University, Yantai, 264099 Shandong China; 3 Department of Gastrointestinal Surgery, The Affiliated Hospital of Qingdao University, Qingdao, 266003 Shandong China; 4 Department of Pathology, Shandong Provincial Hospital Affiliated to Shandong First Medical University, Jinan, 250021 Shandong China; 5 Department of Pathology, The Affiliated Hospital of Qingdao University, Qingdao, 266003 Shandong China; 6 Department of Radiology, Shandong Provincial Hospital Affiliated to Shandong First Medical University, Jinan, 250021 Shandong China

**Keywords:** Magnetic resonance imaging, Radiomics, Tertiary lymphoid structures, Soft tissue sarcoma, Prognosis

## Abstract

**Objectives:**

To develop and validate an MRI-derived radiomics model for the prediction of intratumoral tertiary lymphoid structures (TLSs) status of soft tissue sarcoma (STS) and explore its prognostic value.

**Materials and methods:**

This study retrospectively included 302 patients of three cohorts who underwent surgical resection of STS from two medical centers. Radiomics features were derived for both intratumoral and peritumoral regions from preoperative axial fat-suppressed T2-weighted and T1-weighted imaging sequences. Intratumoral, peritumoral, and combined radiomics models were constructed using a logistic regression algorithm. The area under the receiver operator characteristic curve (AUC) and the DeLong test were utilized to assess and compare the performances of three radiomics models. By applying a linear combination of the chosen features, the Rad-score for the optimal radiomics model was computed.

**Results:**

TLS positivity was identified in 114 (38%) of the 302 patients. No clinical, radiological, or pathological variable was found to show a statistically significant association with TLSs status. The combined radiomics model showed superior performance compared to both the intratumoral and peritumoral models, with an AUC of 0.878 (95% CI 0.812–0.927) in the development cohort, 0.778 (95% CI 0.649–0.876) in the internal validation cohort, and 0.772 (95% CI 0.679–0.850) in the external validation cohort. In the cohort for all patients, the 36-month cumulative PFS rate was 66.1% in the high Rad-score (≥ 0.5) group vs. 37.2% in the low Rad-score group (*p* < 0.05, log-rank test).

**Conclusion:**

An MRI-derived radiomics model could predict intratumoral TLS status in patients with STS and demonstrated a correlation with PFS.

**Critical relevance statement:**

The MRI-derived radiomics model could predict intratumoral TLSs status in patients with STS accurately, which may help to screen patients who will benefit from immunotherapy and have a better prognosis.

**Key Points:**

- Intratumoral tertiary lymphoid structure status in patients with soft tissue sarcomas was accurately predicted by an MRI-derived radiomics model.
- The combined radiomics model showed superior performance compared to both the intratumoral and peritumoral radiomics models.
- Progression-free survival was significantly longer in patients with a high Rad-score (≥ 0.5) in the development, internal validation, and external validation cohorts.

**Graphical Abstract:**

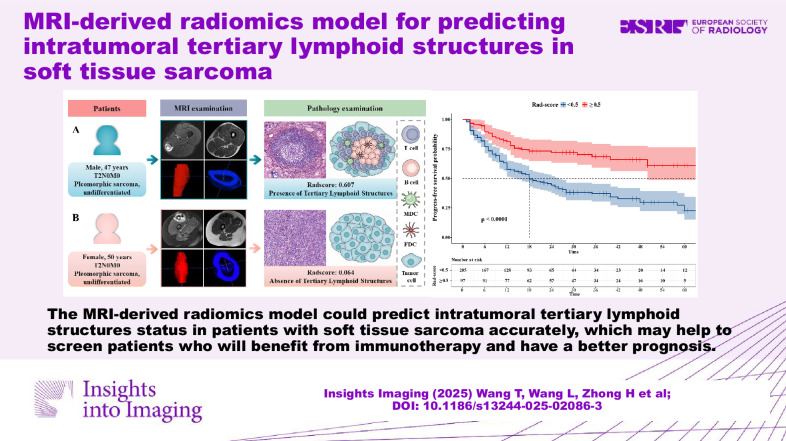

## Introduction

Soft tissue sarcomas (STSs) are a group of malignant interstitial tumors, accounting for about 1% of all malignancies [1–3]. STSs are highly heterogeneous and include over 70 histological subtypes [4]. These tumors have a postoperative recurrence rate of about 33%–50% [3] and a distant metastasis rate of approximately 25%–50% [5]. The elevated risk of local recurrence and distant metastasis led to a poor prognosis for STS patients [3, 5]. Research in this area is becoming increasingly focused on novel immunotherapy for advanced STS because of the limited efficacy of conventional surgery, chemotherapy, and radiotherapy [6]. A primary challenge in the management of STS is how to screen patients who will benefit from immunotherapy through the identification of precise biomarkers [7].

Also known as ectopic lymphoid aggregates, tertiary lymphoid structures (TLSs) develop in non-lymphoid tissues in response to autoimmune disease, chronic inflammation, and cancer [8]. TLSs are described as a zone of CD20^+^ B cells located inside CD3^+^ T cells that is surrounded by separate populations of dendritic cells [9]. TLSs facilitate local production of autoantibodies that perpetuate the pathogenic process [8]. A study by Petitprez et al confirmed that STS patients presenting with TLSs exhibited raised progression-free survival (PFS) time and better immunotherapy response in a multicenter phase 2 clinical trial (SARC028) [10, 11]. TLSs are typically identified in invasive tissue biopsies through hematoxylin-eosin staining, immunohistochemistry, or analysis of gene expression profiles [12]. However, the inherent heterogeneity of TLSs and their dynamic nature over time significantly limit the sensitivity and reliability of these conventional assessment methods. A noninvasive method is urgently needed for assessing TLS status in patients with STS preoperatively.

Given the temporal and spatial heterogeneity of tumors [13], medical imaging has advantages in terms of allowing analysis of the entire tumor and guiding personalized treatment [14]. Emerging as data mining algorithms, radiomics may be seen as a digital biopsy, allowing comprehensive analysis of tumor phenotypes by deriving quantitative variables from medical images [15, 16]. MRI is the standard imaging technique for STS diagnosis in clinical work, which accurately displays the morphological information of the lesions. Previous MRI-derived radiomics studies have provided indirect insights into multiple aspects of STS, including pathological grade, differential diagnosis, evaluation of the therapeutic response, and stratification of the prognosis [3, 5, 17–19].

The objective of this study was to develop an MRI-derived radiomics model for predicting the intratumoral TLS status of STS and to investigate its prognostic value.

## Materials and methods

### Study sample

This study was approved by the Ethics Committee of the Affiliated Hospital of Qingdao University (No. QYFY WZLL 28005). Informed consent was waived due to the retrospective nature of this study, following the principles of the Declaration of Helsinki. This study was registered with the Chinese Clinical Trial Registry (ChiCTR2400082106). This research conformed to CheckList for EvaluAtion of Radiomics Research (CLEAR) [20] and the Machine Learning Evaluation Tools for Imaging Computational Science (METRICS) checklist [21].

Between January 2017 and December 2021, patients who underwent surgical resection and were confirmed as STS by histopathology were retrospectively collected. The data of three cohorts came from two separate hospitals. The inclusion and exclusion criteria are outlined in Supplementary A1. The development cohort (*n* = 142) and internal validation cohort (*n* = 58) were provided by one hospital and the external validation cohort (*n* = 102) by the other hospital. In total, data for 302 patients were included in the study.

### Clinical data and follow-up

Clinical data was extracted from the electronic medical records, including sex, age, location, pathological type, and tumor (T) stage, node (N) stage, and metastasis (M) stage according to the 8th version of the American Joint Committee on Cancer staging system.

Clinical outcomes included disease progression status and PFS. Disease progression was determined when the local recurrence or distant metastasis was discovered by CT, MRI, or PET-CT and confirmed by follow-up or pathological results. PFS was ruled as the period between the date of surgery and the time when the disease progression was diagnosed, the last follow-up, or the date of death, whichever occurred first. For the initial 2 years, follow-up frequency was between 3 and 6 months, and then gradually increased to every 6 months. On December 31, 2023, the follow-up came to an end.

### MRI examination and features

All patients underwent MRI, including axial fat-suppressed T2-weighted imaging (FS-T2WI) and T1-weighted imaging (T1WI). The MRI equipment details were shown in Supplementary A2. Table S1 listed the parameters of the two sequences in detail.

Without knowing the clinical and pathological data of patients, two musculoskeletal radiologists (W.T., with 8 years of diagnosis experience, Y.J., with 19 years of diagnosis experience) evaluated the MRI features independently. The following information was collected: amount (single or multiple); length; FS-T2WI signal (heterogeneous or homogeneous, ≥ 50% tumor volume signal inhomogeneity was defined as heterogeneous); necrosis; margin (obscure or clear); edema; and bone invasion. Any discrepancies in the assessment of MRI features were resolved by discussion between the two radiologists until consensus was reached.

### Histopathological analysis

Without knowing the clinical and radiological data of patients, two musculoskeletal pathologists (Z.Y., with 12 years of diagnostic experience, H.F., with 18 years of diagnostic experience) reviewed all hematoxylin-eosin-stained slides of each patient, respectively. When a discrepancy in assessment occurred, the two pathologists reached a consensus through discussion. In accordance with the maturation stage, TLSs were divided into three classes: lymphoid aggregates (lymphocyte population poorly defined and indistinct); primary lymphoid follicles (characterized by the absence of a germinal center within the lymphoid follicles); and secondary lymphoid follicles (characterized by the presence of a germinal center within the lymphoid follicles). Eleven samples exhibiting high TLS expression were chosen for immunohistochemistry. The methods and aim of immunohistochemistry were stated in Supplementary A3 and Fig. S1. Presence of TLSs was defined as intratumoral TLSs-positive and absence of TLSs as intratumoral TLSs-negative. The French Federation Nationale des Centres de Lutte Contre le Cancer system is defined by three factors, namely, mitotic index, extent of necrosis, and degree of differentiation. Each factor was independently scored and then summed for a histological grade of 1, 2, or 3. The evaluation criterion was the same for the three cohorts.

### Radiomics analysis

One musculoskeletal radiologist (W.T., with 8 years of diagnosis experience) used ITK-SNAP (version 3.8.0, http://www.itksnap.org) software to segment the region-of-interest (ROI) layer by layer, and then the volume of the region (VOI) of tumors was obtained. After segmentation, the Radiomics Intelligent Analysis (RIAS) toolkit (version 1.0.0) [22] was used to create peritumoral masks at a radial distance of 15 mm outside the lesions. Surrounding air, normal tissue, large arteries, and veins were manually excluded, and then the VOI of peri-tumors was obtained. The VOI of tumors and peri-tumors were strictly inspected by another musculoskeletal radiologist (Y.J., with 19 years of diagnosis experience). The Pyradiomics package was employed to extract radiomics features from FS-T2WI and T1WI images. The image preprocessing and transformation methods were detailed in Supplementary A4. 1316 radiomics features of each sequence for each region were extracted, and the feature types were detailed in Supplementary A5.

Preprocessing of the extracted radiomic features included two steps. First, *z*-scores were calculated to achieve the standard normal distribution of image intensities of two MRI sequences. In previous multicenter radiomics studies, the ComBat harmonization method could eliminate variability arising from multiple sources, including differences in scanners, parameters, and protocols, while preserving texture discrimination [3, 23]. Therefore, we used this method as a second step to improve reproducibility.

Three feature-dimension methods were used. First, feature redundancy was minimized by Pearson correlation analysis. When the Pearson correlation coefficient (*r*) exceeded 0.9, the algorithm would randomly eliminate one feature while retaining another. Second, maximum relevance and minimum redundancy chose 40 features with the strongest correlations and the least redundancy. Third, the least absolute shrinkage and selection operator method was used to select features. Next, logistic regression was used to establish three radiomics models: an intratumoral model, which included the radiomics features of intratumoral regions from T1WI and FS-T2WI; a peritumoral model, which included the radiomics features of peritumoral regions (at a radial distance of 15 mm outside the lesions) from T1WI and FS-T2WI; and a combined model, including radiomics features of intratumoral regions and peritumoral regions from T1WI and FS-T2WI.

Using the AUC, sensitivity, and specificity, the ability of the three radiomics models to predict intratumoral TLS status in STS was assessed in the development cohort and tested in the internal and external validation cohorts. The AUCs of the three radiomics models were compared using DeLong’s test. Decision curve and calibration curve analyses were used to evaluate clinical net benefit and model fitness. The Rad-score of the optimal radiomics model was computed by applying a linear combination of the chosen features, with each feature weighted by its corresponding coefficient for each patient.

### Statistical analysis

Using the chi-squared test or Fisher’s exact test, the categorical variables were analyzed. Using the *t*-test, Mann–Whitney *U*-test, or Kruskal–Wallis *H* test, continuous variables were analyzed. Univariate and multivariate analyses were performed to identify clinical, radiological, and pathological parameters that were independent risk factors. The relative risk for each parameter was calculated as the odds ratio. The relationship between TLS status, Rad-score, and PFS was assessed by Cox proportional hazards regression and reported as hazard ratio (HR). Survival according to TLS status and Rad-score was assessed using the Kaplan–Meier method with the log-rank test. MedCalc (version 22.0; MedCalc Software Ltd.), SPSS for Windows (version 26.0; IBM Corp.), and R software (version 3.6.3, www.r-project.org) were used to perform all statistical analyses. A *p*-value of < 0.05 was considered statistically significant.

## Results

### Baseline characteristics

**Fig. 1:**
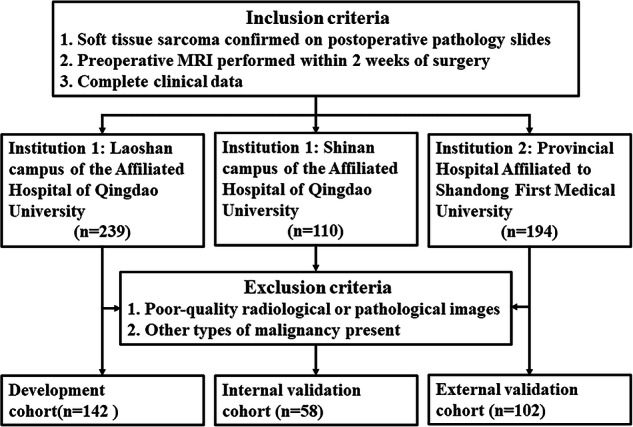
Inclusion and exclusion criteria of the study

Figure 1 shows the enrollment process of patients. One hundred and fourteen patients (38%) were confirmed to be TLS-positive and 188 (62%) to be TLS-negative. There were no significant differences in clinical, radiological, and pathological parameters of STS among the three cohorts except for T stage, French Federation Nationale des Centres de Lutte Contre le Cancer grade, and location (Table 1). Univariate and multivariate analyses did not identify any statistically significant associations of clinical, radiological, or pathological parameters with TLS status (Table 2).

**Table 1:** Clinical, radiological, and pathological characteristics in soft tissue sarcomas

		Development cohort	Internal validation cohort	External validation cohort	*p*-value
TLSs	Positive	55	18	41	0.489
	Negative	87	40	61	
Age (mean ± SD)		58.39 ± 16.29	56.40 ± 15.02	53.75 ± 17.40	0.194
Sex	Male	76	31	50	0.762
	Female	66	27	52	
T stage	T1	21	9	27	0.010
	T2	65	23	36	
	T3	27	13	31	
	T4	29	13	8	
N stage	N0	139	56	98	0.669
	N1	3	2	4	
M stage	M0	133	53	100	0.146
	M1	9	5	2	
FNCLCC	1	23	12	5	0.002
	2	56	21	61	
	3	63	25	36	
Location	Limb	121	47	69	0.009
	Retroperitoneum	14	9	28	
	Head and neck	7	2	5	
Amount	Single	122	51	80	0.187
	Multiple	20	7	22	
Length (mean ± SD)		91.81 ± 58.60	99.88 ± 67.54	87.84 ± 46.89	0.897
T2 signal	No	103	49	79	0.188
	Yes	39	9	23	
Necrosis	No	66	17	40	0.075
	Yes	76	41	62	
Margin	Obscure	52	21	24	0.074
	Clear	90	37	78	
Edema	No	13	6	16	0.275
	Yes	129	52	86	
Bone invasion	No	125	50	81	0.171
	Yes	17	8	21	

*TLSs* Tertiary lymphoid structures, *FNCLCC* French Federation Natinale des Centres de Lutte Contre le Cancer

**Table 2:** Univariate and Multivariate logistic analysis of clinical and MRI characteristics

Variable	Univariate		Multivariate	
	OR (95% CI)	*p*-value	OR (95% CI)	*p*-value
Age	1.00 (0.98–1.02)	0.888	1.00 (0.98–1.03)	0.757
Sex	1.36 (0.69–2.69)	0.376	1.46 (0.70–3.03)	0.317
T stage	1.01 (0.71–1.42)	0.97	0.99 (0.51–1.93)	0.985
N stage	0.79 (0.07–8.89)	0.847	1.10 (0.08–14.51)	0.945
M stage	0.43 (0.09–2.16)	0.306	0.53 (0.09–3.16)	0.482
FNCLCC	0.73 (0.46–1.17)	0.194	0.70 (0.39–1.25)	0.222
Location	1.02 (0.52–1.98)	0.958	0.88 (0.40–1.93)	0.756
Amount	0.35 (0.11–1.10)	0.073	0.33 (0.10–1.12)	0.075
Length	1.00 (1.00–1.01)	0.796	1.00 (0.99–1.01)	0.867
T2 signal	0.85 (0.39–1.82)	0.67	0.75 (0.26–2.18)	0.601
Necrosis	0.84 (0.43–1.66)	0.62	0.81 (0.33–1.96)	0.638
Margin	0.90 (0.45–1.80)	0.759	0.80 (0.37–1.73)	0.565
Edema	1.01 (0.31–3.27)	0.983	1.12 (0.30–4.16)	0.861
Bone invasion	0.85 (0.29–2.44)	0.757	0.91 (0.25–3.39)	0.89

*OR* odd ratio, *CI* confidence interval, *FNCLCC* French Federation Natinale des Centres de Lutte Contre le Cancer

The pathological types of STS are shown for all 302 patients in the three cohorts in Table 3. The TLSs status for each pathological type is shown in Table S2. When pathological types contained more than 10 cases, dedifferentiated liposarcoma (67%), undifferentiated pleomorphic sarcoma (54%), and well-differentiated liposarcoma (50%) had the highest TLS positivity rates.

**Table 3:** Pathological types of soft tissue sarcomas

Pathological type	Development cohort	Internal validation cohort	External validation cohort	Total
Pleomorphic sarcoma, undifferentiated	25	15	10	50
Myxofibrosarcoma	20	8	11	39
Myxoid liposarcoma	16	7	11	34
Fibrosarcoma, others	18	4	10	32
Uncertain differentiation, others	10	5	17	32
Leiomyosarcoma	11	7	11	29
Liposarcoma, well-differentiated	15	6	5	26
Dedifferentiated liposarcoma	8	1	12	21
Synovial sarcoma	7	3	4	14
Rhabdomyosarcoma	2	0	6	8
Alveolar soft part sarcoma	5	2	1	8
Liposarcoma, others	2	0	4	6
Malignant peripheral nerve sheath tumor	2	0	0	2
Angiosarcoma	1	0	0	1
Total	142	58	102	302

### Performance of the radiomics model

**Fig. 2:**
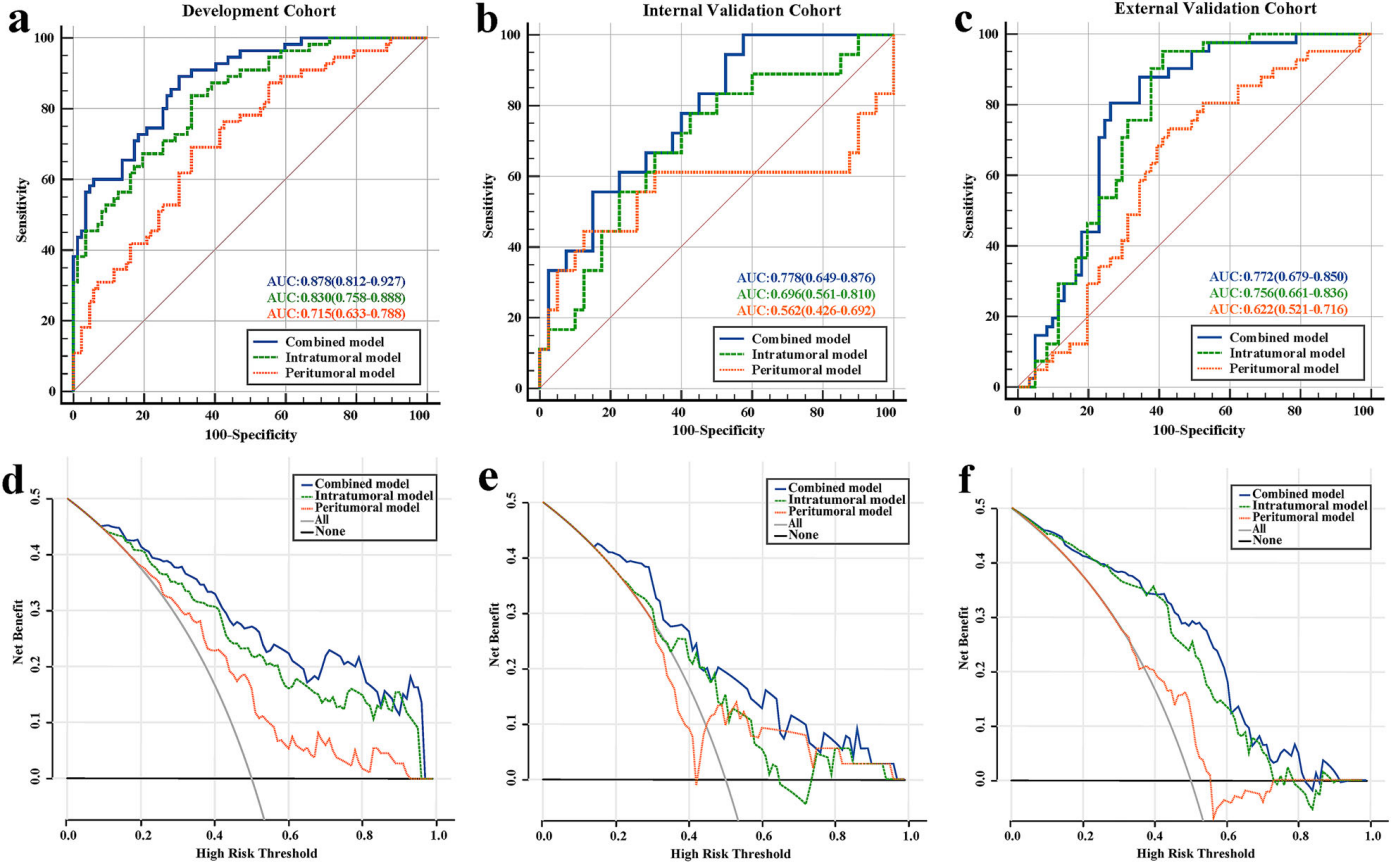
Receiver-operating characteristic and decision curve analyses for the intratumoral, peritumoral, and combined radiomics models in the development (**a**, **d**), internal validation (**b**, **e**), and external validation (**c**, **f**) cohorts

The predictive performance of the combined model for assessing TLSs status was significantly superior to that of both the intratumoral and peritumoral models, with an AUC of 0.878 (95%CI 0.812–0.927) in the development cohort, 0.778 (95%CI 0.649–0.876) in the internal validation cohort, 0.772 (95%CI 0.679–0.850) in the external validation cohort (Table 4 and Fig. 2). Three radiomics models’ input features and coefficients were shown in Tables S3. The results of Delong’s test are provided in Supplementary A6. The calibration curve showed strong alignment, indicating that the combined model was well-fitted across the three cohorts (Fig. S2). Decision curve analysis demonstrated that the combined model provided a superior clinical net benefit compared to the other two radiomics models (Fig. 2).

**Table 4:** Performance of radiomics models

Radiomics models	Development cohort			Internal validation cohort			External validation cohort		
	AUC (95% CI)	Sensitivity	Specificity	AUC (95% CI)	Sensitivity	Specificity	AUC (95% CI)	Sensitivity	Specificity
Intratumoral model	0.830 (0.758–0.888)	0.836	0.667	0.696 (0.561–0.810)	0.778	0.575	0.756 (0.661–0.836)	0.951	0.59
Peritumoral model	0.715 (0.633–0.788)	0.691	0.667	0.562 (0.426–0.692)	0.444	0.875	0.622 (0.521–0.716)	0.732	0.574
Combined model	0.878 (0.812–0.927)	0.891	0.701	0.778 (0.649–0.876)	0.691	0.667	0.772 (0.679–0.850)	0.805	0.738

*AUC* area under the receiver operating characteristic curve, *CI* confidence interval

**Fig. 3:**
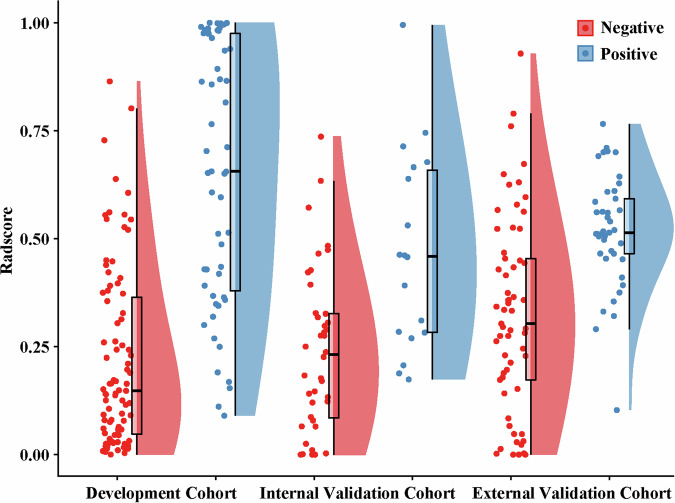
Cloud and rain plots comparing Rad-scores between the TLS-negative and TLS-positive groups across the development, internal validation, and external validation cohorts. TLSs, tertiary lymphoid structures

**Fig. 4:**
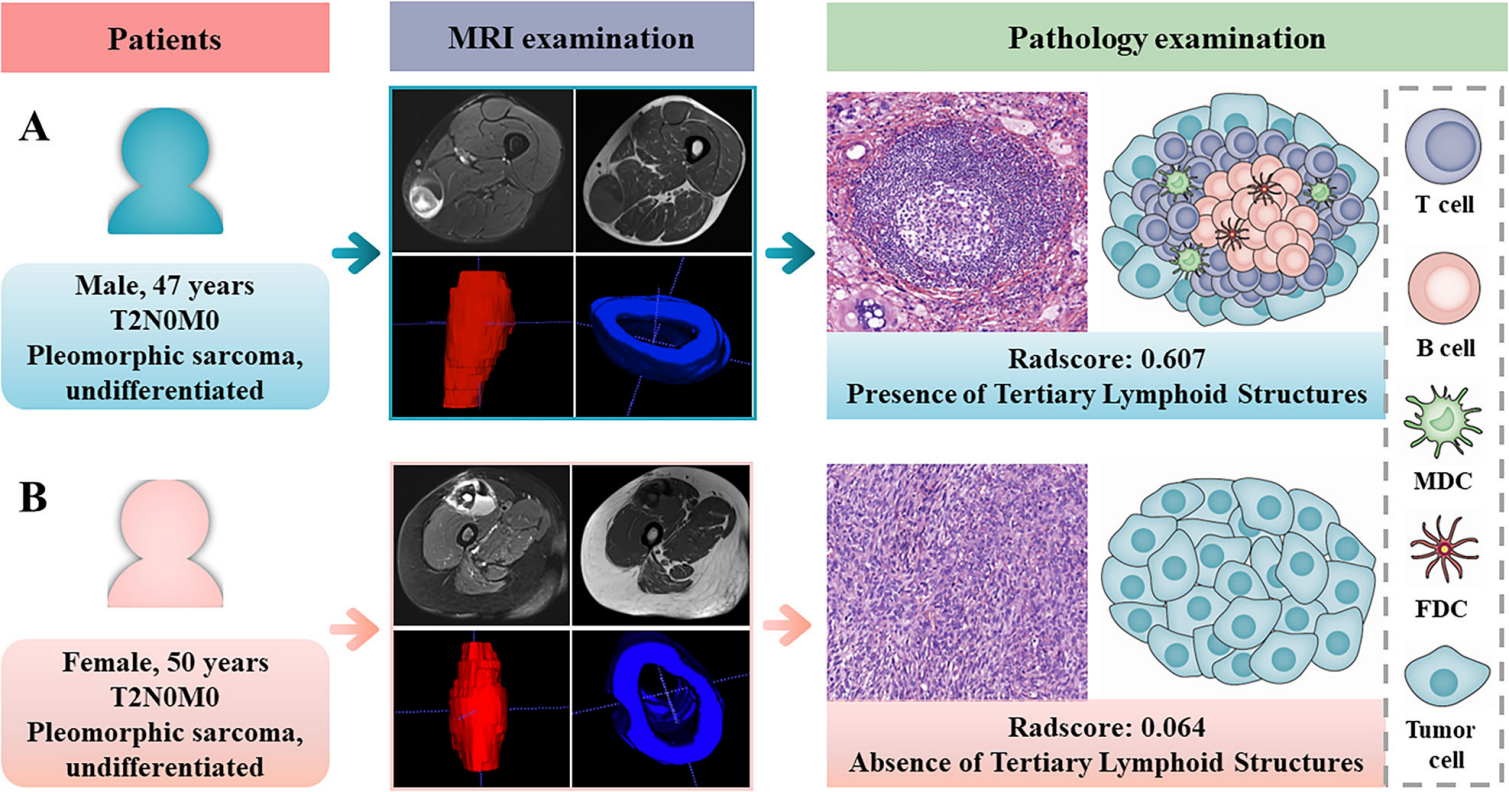
Representative cases from the TLSs-positive and TLSs-negative groups. The Rad-score of patient **A** was 0.607, suggesting a TLS-positive status. Patient **A** had a postoperative follow-up of 43 months without recurrence or distant metastasis. The Rad-score of patient **B** was 0.064, suggesting TLS-negative status. Patient **B** experienced recurrence at 10 months postoperatively. TLSs, tertiary lymphoid structures

Based on the combined model, the Rad-score was calculated for each patient. The logistic regression function was detailed in Supplementary A7. Patients who were TLSs-positive exhibited significantly higher Rad-score compared to those who were TLSs-negative in all three cohorts, with all *p*-values being < 0.001 (Fig. 3). Figure 4 shows representative cases from the TLSs-positive and TLSs-negative groups.

### Associations of TLS status and Rad-score with PFS

**Fig. 5:**
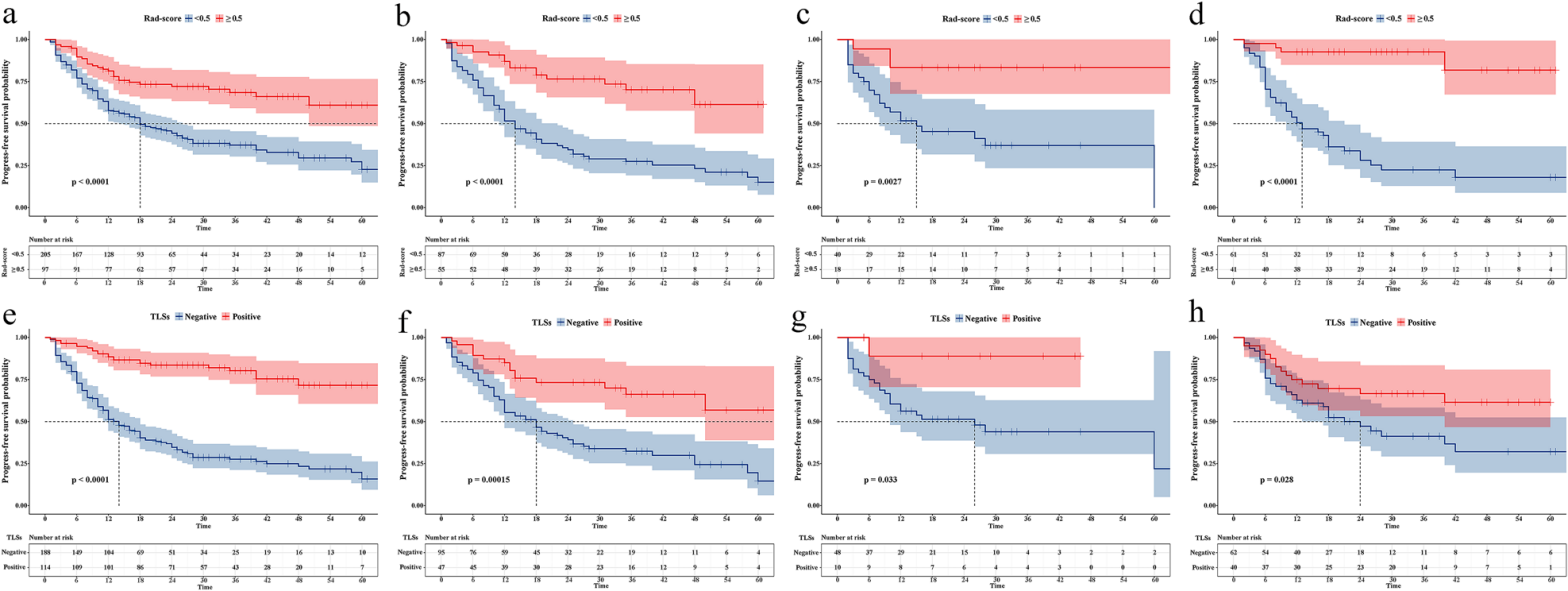
Kaplan–Meier curves depicting progression-free survival (PFS) in soft tissue sarcoma (STS) patients when stratified by the Rad-score (cut-off 0.5) for all patients (**a**) and the development (**b**), internal validation (**c**), and external validation (**d**) cohorts. Kaplan–Meier curves depicting PFS in patients with STS stratified by TLS status in all patients (**e**) and in the development (**f**), internal validation (**g**), and external validation (**h**) cohorts. TLSs, tertiary lymphoid structures

The Cox proportional regression model revealed significant associations of TLS status and the Rad-score with PFS (HR 0.183, 95% CI 0.118–0.286 and HR 0.411, 95% CI 0.279–0.606; both *p* < 0.05). Kaplan–Meier survival curves for all patients, development, internal validation, and external validation cohorts revealed significant statistical differences in PFS between the TLSs-positive group and TLSs-negative group and between the high Rad-score (≥ 0.5) group and the low Rad-score group (all *p* < 0.05, log-rank test) (Fig. 5). In the cohort for all patients, the 6-, 12-, 24-, 36-, and 48-month cumulative PFS rate was 94.7%, 88.5%, 83.6%, 75.4%, 71.6% in TLSs-positive group vs. 72.8%, 51.3%, 34.7%, 27.6%, 23.4% in TLSs-negative group, respectively. The 6-, 12-, 24-, 36-, and 48-month cumulative PFS rate was 89.6%, 81.3%, 72.1%, 66.1%, 61.0% in the high Rad-score group vs. 77.0%, 57.8%, 44.3%, 37.2%, 29.6% in the low Rad-score group, respectively.

## Discussion

This study revealed a correlation between the presence of intratumoral TLSs and a favorable prognosis in patients with STS, which is consistent with previous research [24]. STS with various pathological subtypes frequently exhibit an atypical manner, and their responses to immune checkpoint blockade therapies differ significantly [11, 25]. A recent retrospective study by Petitprez et al revealed an association between the presence of TLSs in STS and the response to pembrolizumab [10]. Pembrolizumab has been demonstrated to have significant clinical efficacy in patients with dedifferentiated liposarcoma and undifferentiated pleomorphic sarcoma [11]. In our study, dedifferentiated liposarcoma (67%) and undifferentiated pleomorphic sarcoma (54%) had the highest TLS positivity rates of all the pathological subtypes; this finding provides some evidence in support of a relationship between TLS status and the efficacy of immunotherapy. TLS status has the potential to be a biomarker of the prognosis of STS and the efficacy of immunotherapy.

We retrospectively analyzed the medical data for 302 patients with STS. No significant predictors of TLS status were identified in univariate and multivariate analyses. This finding suggested that the ability of low latitudinal clinical, radiological, and pathological data to predict TLS status in STS is limited. Therefore, there is a need for a more efficient and accurate method for clinical assessment of TLS status in patients with STS. By transforming conventional medical images into analyzable quantitative data, radiomics facilitates evaluation of tumor heterogeneity in terms of histopathological grade, therapeutic effects, and the prognosis in patients with STS [3, 26, 27]. Radiomics has recently been used to predict intratumoral TLS status in solid tumors [28, 29]. Xu et al [28] constructed an MRI-derived radiomics signature for predicting intratumoral TLS status non-invasively and preoperatively in intrahepatic cholangiocarcinoma patients and reported AUCs of 0.81 and 0.84 in the internal and external validation groups. Li et al [29] built a radiomics nomogram to predict intratumoral TLS status in breast cancer patients and found an AUC of 0.749 in the test set. These findings indicate that radiomics has a strong ability to predict intratumoral TLS status. In our study, the combined radiomics model achieved an AUC of 0.778 in the internal validation cohort and 0.772 in the external validation cohort by using logistic regression, which demonstrates robust performance with small sample sizes, effectively prevents overfitting, and maintains both strong clinical interpretability and practical utility [30].

Among the selected radiomics features for constructing a combined model, “exponential_ngtdm_Complexity” contributed the most with a coefficient of 1.563, which reflects the complexity of an image’s texture by analyzing the differences in gray-tone values between neighboring pixels using an exponential function. This may be because the distribution of multiple immune cells (B cells, T cells, and dendritic cells) within TLSs, combined with microvascular proliferation, amplifies local gray-level intensity variations on imaging. This elevated complexity signifies an immunologically active state, which is subsequently associated with prolonged PFS [10]. In the future, this model could be used widely in the clinical setting for the prediction of TLS status in patients with STS, and spatial transcriptomics should be integrated to further delineate molecular signatures within specific TLS regions.

Tumor biology studies have shown that a large number of cytokines and growth factors secreted by the tumor immune microenvironment can not only induce hypoxia and angiogenesis but also contribute to the development and metastasis of tumors [31, 32]. Peritumoral regions, namely, regions surrounding the tumor, would also contain valuable complementary information about tumors [33]. The principle for using peritumoral radiomics to predict the status of biomarkers, like HER-2 and Ki-67 status, has been proven in recent studies [34, 35]. Fan et al [34] developed a combined radiomic signature that integrates intratumoral and peritumoral features to predict the HER-2 and Ki-67 status of breast tumors; this signature had AUCs of 0.848 and 0.800, respectively, in the validation cohort. Zhu et al [35] showed that peritumoral zones provide data that supplement the information provided by intratumoral regions for predicting Ki-67 proliferation status in glioblastoma. We have proposed an MRI-derived combined radiomics model incorporating 8 intratumoral radiomics features and 5 peritumoral radiomics features, demonstrating superior predictive value for assessing TLS status in patients with STS compared to models using solely intratumoral or peritumoral features (AUC 0.778 vs 0.696 and 0.562, respectively, in the internal validation cohort; 0.772 vs 0.756 and 0.622 in the external validation cohort). Our combined radiomics model enabled accurate and convenient preoperative assessment of TLS status in patients with STS, thereby facilitating the formulation of precise strategies for clinical decision-making.

This study also demonstrated that the combined radiomics model allowed risk stratification for PFS in patients with STS, suggesting that accurate prediction of TLS status is closely associated with the prognostic evaluation. The response rate was reported to be higher and the PFS longer in patients with STS characterized by a TLSs-specific gene expression signature than in patients wth other types of STS [36]. Our combined radiomics model has the potential to select patients who may benefit from immunotherapy and allow an individualized treatment plan, which is particularly beneficial in these patients.

Several notable limitations of this study warrant attention. First, the retrospective study exists with inevitable selection bias. However, the radiomics models were validated in two separate cohorts, confirming the reliability of our data. Second, intratumoral and peritumoral zones were delineated by manual contouring, which is both labor-intensive and time-consuming. Automated segmentation is needed to streamline this procedure in further research. Third, the ComBat compensation method was applied to maintain effective predictive features while mitigating the adverse effects arising from variances in scanners and protocols across two centers. Fourth, the radiomics models were only based on the MR plain scan sequence. The enhanced T1-weighted sequences should be included in future studies to further improve the model’s performance. Prospective data should now be collected from larger numbers of patients from more centers to confirm the TLSs status predictive ability of our radiomics model.

In conclusion, a combined radiomics model based on intratumoral and peritumoral features could act as a valuable noninvasive tool for predicting intratumoral TLS status in STS patients preoperatively and non-invasively. TLS status was found to be significantly correlated with PFS.

## Supplementary information

ELECTRONIC SUPPLEMENTARY MATERIAL

## Data Availability

The data can be obtained from the corresponding author upon reasonable request approved by the Institutional Review Board of all registered centers. Because raw images and follow-up data contain sensitive information that could compromise patient privacy, they are not made public.

## Abbreviations

AUC: Area under the receiver operator characteristic curve
FS-T2WI: Fat-suppressed T2-weighted imaging
HR: Hazard ratio
M stage: Metastasis stage
N stage: Node stage
PFS: Progression-free survival
STSs: Soft tissue sarcomas
T stage: Tumor stage
T1WI: T1-weighted imaging
TLSs: Tertiary lymphoid structures
VOI: Volume of interest
