# Supplementary material for: MRI-derived radiomics model for predicting intratumoral tertiary lymphoid structures in soft tissue sarcoma
Source: Insights Imaging. 2025 Sep 23;16:201. doi: 10.1186/s13244-025-02086-3 (PMC12457251; doi:10.1186/s13244-025-02086-3)
Supplement: Supplementary file 1 — ELECTRONIC SUPPLEMENTARY MATERIAL [file 13244_2025_2086_MOESM1_ESM.pdf]

MRI-derived radiomics model for predicting intratumoral tertiary lymphoid structures in soft tissue sarcoma

ELECTRONIC SUPPLEMENTARY MATERIAL

Supplementary A1. Study inclusion and exclusion criteria

The inclusion criteria were as follows: (a) STS confirmed on postoperative pathology slides; (b) preoperative MRI performed within 2 weeks of surgery; and (c) complete clinical data. The exclusion criteria were (a) poor-quality radiological or pathological images and (b) other types of malignancy present. STS, soft tissue sarcoma

Supplementary A2. Equipment used for MRI

The MRI scanners used were the Philips Achieva 1.5T (Philips Healthcare, Andover, MA), the GE HDx 1.5T, 3.0T (GE Medical Systems, Milwaukee, WI), and the Siemens Magnetom Skyra 3.0T and Prisma 3.0T (Siemens Healthcare, Erlangen, Germany).

Supplementary A3. The methods and aim of immunohistochemistry

The criteria to define TLSs-positive status was the labeled CD3, CD20, and CD21 expression within the cluster-like lymphocyte structures concurrently. TLSs were deemed mature when high levels of CD20 and CD21 expression were observed in the germinal centers. Simultaneously, two pathologists marked all TLSs in the HE staining slices of the eleven samples. Immunohistochemistry was employed to conform the presence of true TLSs and to assess the consistency of two pathologists' results. Upon evaluation, the majority of the results were found to be consistent.

Supplementary A4. Image preprocessing and transformation methods

The wavelet, square, square root, logarithm, exponential, and gradient methods in the analysis software were used for preprocessing and image transformation.

Supplementary A5. Types of features extracted

The radiomics features included shape, first-order statistics, and the gray level co-occurrence matrix (GLCM), gray level dependence matrix (GLDM), gray level size zone matrix (GLSZM), gray level run length matrix (GLRLM), and neighborhood gray tone difference matrix (NGTDM).

Supplementary A6. Results of Delong's test

Significant differences in AUC values were identified among the following pairs of models: (1) In the development cohort, combined model vs. intratumoral model, P=0.024; combined model vs. peritumoral model, P<0.001; and intratumoral model vs. peritumoral model, P=0.013. (2) In the internal validation cohort, combined model vs. peritumoral model, P=0.018. (3) In the external validation cohort, combined model vs. peritumoral model, P=0.008; and intratumoral model vs. peritumoral model, P=0.046. No significant differences in the AUC were identified among the pairs of models described below: (1) In the internal validation cohort, combined model vs. intratumoral model, P=0.241; and intratumoral model vs. peritumoral model, P=0.266. (2) In the external validation cohort, combined model vs. intratumoral model, P=0.662.

Supplementary A7. The logistic regression function

Radscore = (T_T1_exponential_ngtdm_Busyness×0.253)-(T_T1_square_firstorder_Skewness×0.538)+ +(T_T2_wavelet.HHL_gldm_LargeDependenceLowGrayLevelEmphasis×0.279)+(T_T2_wavelet.HLL_firstorder_Skewness×0.776)-(T_T2_gradient_glcm_MCC×0.205)+(T_T2_original_shape_Elongation×1.039)+(T_T2_logarithm_gldm_LargeDependenceLowGrayLevelEmphasis×0.391)+(T_T2_logarithm_firstorder_Skewness×0.718)+(C_T2_logarithm_glcm_InverseVariance×0.243)-(C_T1_squareroot_firstorder_Minimum×0.835)+(C_T1_exponential_ngtdm_Complexity×1.536)-(C_T1_gradient_gldm_SmallDependenceLowGrayLevelEmphasis×0.322)+(C_T2_wavelet.LHL_gldm_SmallDependenceLowGrayLevelEmphasis×0.730) -0.561

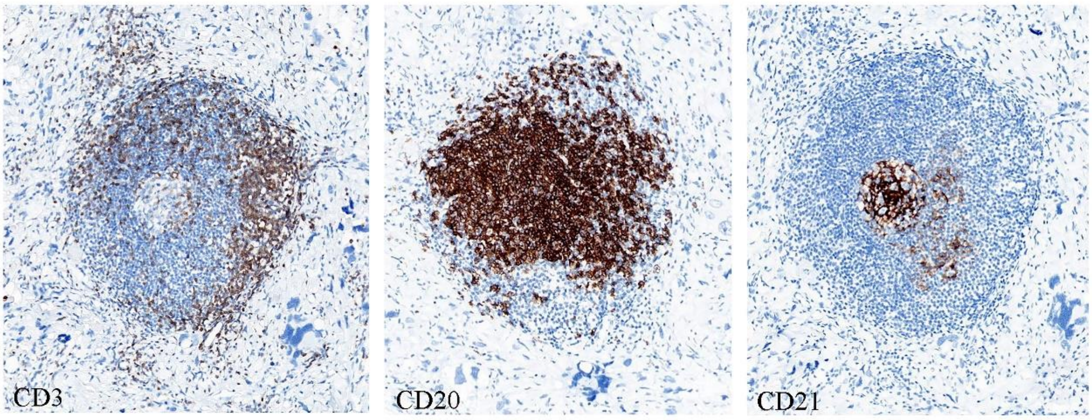

Figure S1: Immunohistochemistry staining images from a 62-year-old patient with soft tissue sarcoma exhibiting high expression of tertiary lymphoid structures (TLSs). The staining included markers for CD3, CD20, and CD21. CD3-labeled T cells were predominantly situated at the periphery of the TLSs, while CD20-labeled B cells showed high expression in the central region. Additionally, CD21-labeled dendritic cells were observed forming a network-like structure within the center of the TLSs.

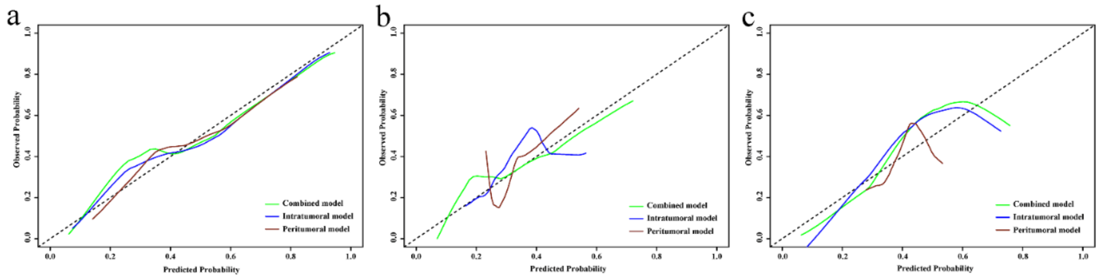

Figure S2: Calibration curves for the combined model, intratumoral model, and peritumoral model in the development (a), internal validation (b), and external validation (c) cohorts.

Table S1. Parameters of MRI sequences

	T1WI	FS-T2WI
TR (ms)	500-600	2400-4500
TE (ms)	10-15	70-120
Slice thickness (mm)	4-5	
Slice spacing (mm)	1	
Matrix	320×320	
FOV (mm)	200-400	

TR, repetition time; TE, echo time; FOV, field of view

Table S2. TLSs status of different pathological types in soft tissue sarcomas

Pathological type	TLSs Positive	TLSs Negative	Total
Pleomorphic sarcoma, undifferentiated	27(54%)	23(46%)	50
Myxofibrosarcoma	14(36%)	25(64%)	39
Myxoid liposarcoma	4(12%)	30(88%)	34
Fibrosarcoma, others	12(38%)	20(62%)	32
Uncertain differentiation, others	11(34%)	21(66%)	32
Leiomyosarcoma	9(31%)	20(69%)	29
Liposarcoma, well-differentiated	13(50%)	13(50%)	26
Dedifferentiated liposarcoma	14(67%)	7(33%)	21
Synovial sarcoma	0(0)	14(100%)	14
Rhabdomyosarcoma	5(62%)	3(38%)	8
Alveolar soft part sarcoma	1(12%)	7(88%)	8
Liposarcoma, others	4(67%)	2(33%)	6
Malignant peripheral nerve sheath tumour	0(0)	2(100%)	2
Angiosarcoma	0(0)	1(100%)	1
Total	114	188	302

TLSs, Tertiary lymphoid structures.

Table S3. Input features of radiomics models

Radiomics models	Input features	Coefficients
Intratumoral model	T_T1_original_firstorder_Skewness	-0.335
	T_T1_exponential_ngtdm_Busyness	0.352
	T_T2_logarithm_gldm_LargeDependenceLowGrayLevelEmphasis	0.321
	T_T2_wavelet.HHL_gldm_LargeDependenceLowGrayLevelEmphasis	0.184
	T_T2_wavelet.HLL_firstorder_Skewness	-0.695
	T_T2_gradient_glcm_MCC	-0.442
	T_T2_original_shape_Elongation	1.047
	T_T2_logarithm_glszm_GrayLevelNonUniformityNormalized	0.362
	T_T2_logarithm_firstorder_Skewness	0.602
Peritumoral model	C_T1_gradient_gldm_SmallDependenceLowGrayLevelEmphasis	-0.589
	C_T2_wavelet.LHL_glszm_SmallAreaLowGrayLevelEmphasis	0.225
	C_T2_logarithm_glcm_InverseVariance	0.614
Combined model	T_T1_square_firstorder_Skewness	-0.538
	T_T1_exponential_ngtdm_Busyness	0.253
	T_T2_wavelet.HHL_gldm_LargeDependenceLowGrayLevelEmphasis	0.279
	T_T2_wavelet.HLL_firstorder_Skewness	0.776
	T_T2_gradient_glcm_MCC	-0.205
	T_T2_original_shape_Elongation	1.039
	T_T2_logarithm_gldm_LargeDependenceLowGrayLevelEmphasis	0.391
	T_T2_logarithm_firstorder_Skewness	0.718
	C_T2_logarithm_glcm_InverseVariance	0.243
	C_T1_squareroot_firstorder_Minimum	-0.835
	C_T1_exponential_ngtdm_Complexity	1.536
	C_T1_gradient_gldm_SmallDependenceLowGrayLevelEmphasis	-0.322
	C_T2_wavelet.LHL_gldm_SmallDependenceLowGrayLevelEmphasis	0.730
